# ZIKV – CDB: A Collaborative Database to Guide Research Linking SncRNAs and ZIKA Virus Disease Symptoms

**DOI:** 10.1371/journal.pntd.0004817

**Published:** 2016-06-22

**Authors:** Victor Satler Pylro, Francislon Silva Oliveira, Daniel Kumazawa Morais, Sara Cuadros-Orellana, Fabiano Sviatopolk-Mirsky Pais, Julliane Dutra Medeiros, Juliana Assis Geraldo, Jack Gilbert, Angela Cristina Volpini, Gabriel Rocha Fernandes

**Affiliations:** 1 Biosystems Informatics and Genomics Group, René Rachou Research Center (CPqRR-FIOCRUZ), Belo Horizonte, Minas Gerais, Brazil; 2 Department of Ecology and Evolution, The University of Chicago, Chicago, Illinois, United States of America; 3 Argonne National Laboratory, Institute for Genomic and Systems Biology, Argonne, Illinois, United States of America; 4 Department of Surgery, The University of Chicago, Chicago, Illinois, United States of America; Colorado State University, UNITED STATES

## Abstract

**Background:**

In early 2015, a ZIKA Virus (ZIKV) infection outbreak was recognized in northeast Brazil, where concerns over its possible links with infant microcephaly have been discussed. Providing a causal link between ZIKV infection and birth defects is still a challenge. MicroRNAs (miRNAs) are small noncoding RNAs (sncRNAs) that regulate post-transcriptional gene expression by translational repression, and play important roles in viral pathogenesis and brain development. The potential for flavivirus-mediated miRNA signalling dysfunction in brain-tissue development provides a compelling hypothesis to test the perceived link between ZIKV and microcephaly.

**Methodology/Principal Findings:**

Here, we applied *in silico* analyses to provide novel insights to understand how Congenital ZIKA Syndrome symptoms may be related to an imbalance in miRNAs function. Moreover, following World Health Organization (WHO) recommendations, we have assembled a database to help target investigations of the possible relationship between ZIKV symptoms and miRNA-mediated human gene expression.

**Conclusions/Significance:**

We have computationally predicted both miRNAs encoded by ZIKV able to target genes in the human genome and cellular (human) miRNAs capable of interacting with ZIKV genomes. Our results represent a step forward in the ZIKV studies, providing new insights to support research in this field and identify potential targets for therapy.

## Author Summary

The potential interaction between ZIKA Virus (ZIKV) infection and infant brain defects still has no explanatory mechanism. The mechanism of action for several other viruses have been described, and are often related to the control or production of sncRNA (small noncoding RNA) molecules, which regulate the expression of several human genes. Based on this knowledge, we have predicted miRNAs (a kind of sncRNA) encoded by ZIKV genomes, and identified human miRNAs that may be able to interact with human genes related to microcephaly. To help support further investigation, we have created a searchable database for ZIKV miRNAs that may mimic human molecules and possibly interfere with the human gene expression; or to search for human sncRNA molecules that could be recruited by the ZIKV genome, and influence function. This database could aid investigators in elucidating the role played by sncRNAs in human ZIKV infection, and be used to identify novel therapeutic targets.

## Introduction

Zika virus (ZIKV) is an emerging mosquito-borne flavivirus, first isolated in 1947 from the serum of a pyrexial rhesus monkey caged in the Zika Forest (Uganda/Africa) [1]. In 2007, ZIKV was reported linked to an outbreak of relatively mild disease, characterized by rash, arthralgia, and conjunctivitis on Yap Island, in the western Pacific Ocean [2]. In 2015, ZIKV circulated in the Americas, probably introduced through Easter Island (Chile) by French Polynesians [3], where concerns over its links with infant microcephaly have been raised. MicroRNAs (miRNAs) are small noncoding RNAs (sncRNAs) that regulate post-transcriptional gene expression by translational repression. It is estimated that more than 60% of human protein-coding genes are likely to be under the control of miRNAs [4]. Two hypotheses exist as to how miRNAs could influence ZIKV/human-host interaction. First, the virus could transcribe miRNAs that provide benefits associated with cellular and viral gene expression (e.g. Herpesvirus, Polyomavirus, Ascovirus, Baculovirus, Iridovirus, Adenovirus families) [5, 6]. RNA retrovirus miRNAs are transcribed through RNA polymerase III (pol III), instead of pol II. Virus-encoded miRNAs support persistent infections through subtle modulation of gene expression, leading to prevention of host cell death, evasion of the host immune system and regulation of the latent-lytic switch [7]. Second, retrovirus genomes may directly interact with cellular miRNAs to enhance viral replication potential [5]. By recruiting/exploiting cellular miRNAs, an RNA virus can disturb the regulation of host gene expression, which can trigger molecular disease. In order to provide a theoretical background for future experimental verification of these hypotheses, the ZIKV collaborative database (ZIKV-CDB) was assembled. This enables, (i) searching for predicted ZIKV miRNAs mimicking human miRNAs [searching criteria includes: “Gene name”, “Gene Symbol” or “Ensembl ID”] (hypothesis 1); and (ii) searching for human miRNAs with possible binding-sites to the ZIKV genomes (hypothesis 2).

## Materials and Methods

The ZIKV-CDB comprises miRNAs predicted using HHMMiR [8] for all complete ZIKV genomes currently available at the GenBank (February, 2016—http://www.ncbi.nlm.nih.gov). Hairpin prediction was performed for all *de novo* miRNAs using previously predicted RNA secondary structure [9], and mature miRNAs were delineated with PHDcleav [10]. Potential human genome (Ensembl GRCh37) target sites for the predicted ZIKV miRNAs were detected with miRanda [11] using default parameters (minimum score = 140; minimum energy = 1). Also, all mature human miRNA sequences from miRBase Sequence Database (Release 21—http://www.mirbase.org) were retrieved and mapped against the available ZIKV genomes using miRanda [11] with default parameters, to keep only those miRNAs with a minimum complementarity to ZIKV genomes [at least with complementarity to the miRNA seed region (6–10 nt) of the miRNA]. The ZIKV-CDB is publicly available through a web interface at http://zikadb.cpqrr.fiocruz.br.

### Database construction strategy

The ZIKA Virus Collaborative Database (ZIKV-CDB) was constructed based on two strategies. The first one consists in identifying ZIKA virus (ZIKV) microRNA (miRNA) molecules that may affect human gene expression. The second strategy consists in identifying human microRNA molecules that may be recruited by the ZIKV genome. Our search included the full set of cDNA sequences of the human genome available on the Ensemble database (release 83) [29] for targets of the predicted ZIKV mature miRNA molecules, using the software miRanda [11].

### Mature microRNA prediction

The mature miRNA sequences were predicted using a pipeline based on three steps. The first step uses the tool RNAfold [9] to compute the minimum free energy and to predict the secondary structures based on Zika virus genome cDNA sequences (see ZIKV genomes accession number section). The second step uses the predicted secondary structures to identify the hairpins formed by miRNA precursors using the HHMMiR workflow [8]. The third step uses the software PHDcleav [10] to identify cleavage sites of the Dicer human enzyme in the hairpin structures to generate the sequences of the mature miRNA. A fasta file containing all predicted precursor and mature sequences of the nine ZIKV-encoded miRNAs is provided in the S1 and S2 Datasets, respectively.

### Detection of microRNA target genes

The miRNA molecules suppress post-transcriptional gene expression through physical interaction with the messenger RNA (mRNA) [30]. To detect miRNA target gene candidates, we used the approach presented by the software miRanda (11), which employs the local alignment of the miRNA and mRNA molecules combined with the information of minimum free energy of each nucleotide match of RNA-RNA duplexes. The free energy (ΔG) of optimal strand-strand interaction for each match of alignment was determined using the Vienna package [8]. A detailed table containing the miRNA identifiers from ZIKV, target Ensembl transcripts, total score, total energy, maximum score per alignment, maximum energy per alignment, strand, length of the miRNA, length of the target, and the alignment positions is provided as S3 Dataset. Similarly, a detailed table containing the miRNA identifiers from human, target region in the ZIKV genomes, total score, total energy, maximum score per alignment, maximum energy per alignment, strand, length of the miRNA, length of the target, and the alignment positions is provided as S4 Dataset.

### ZIKV genomes GenBank accession numbers

Kedougou Virus (NC_012533 and AY632540) Bagaza Virus (NC_012534 and AY632545); ZIKA Virus isolated from Uganda (LC002520, NC_012532, AY632535), from Central Africa Republic (KF268949, KF268948 and KF268950), from Brazil (KU527068, KU321639, KU365778, KU365779, KU365777 and KU365780), Martinique (KU647676), French Polynesia (KJ776791), Haiti (KU509998) and Puerto Rico (KU501215).

### Phylogenetic analysis

All recovered ZIKV genome sequences were aligned using the software ClustalW7. Further, the phylogenetic tree was constructed using the online tool Itol: Interactive Tree of Life [12], applying the Neighbor-joining method, with 100 bootstrap repetitions.

## Results and Discussion

We introduce the ZIKV-CDB, a collaborative database encompassing both, predicted miRNAs encoded by ZIKV genomes that could potentially target the human genome, and cellular (human) miRNAs with sequence complementary to ZIKV genomes. This knowledgebase should facilitate researchers when exploring targets that may affect the expression of genes associated with microcephaly and other neurodevelopmental syndromes caused by ZIKV infection. The chosen method for predict ZIKV-encoded miRNAs was based on a previously published benchmark [13], which shows that among the evaluated tools, miRanda had the highest sensitivity for predicting miRNAs, providing more targets for validation. To increase the effectiveness of this strategy, further analysis using genome sequences of other viruses with experimentally validated virally encoded miRNAs should be explored as positive controls. In contrast to previous reports [14, 15], the miRNAs identified here are located in the ZIKV polyprotein coding region. Recently, a study using a similar approach identified miRNAs located in the CDS region of Ebola Virus [16].

Examples of genes predicted to be targeted by miRNAs and previously validated as having a potential link to neurological disorders include the peroxisomal biogenesis factor 26 gene (PEX26), the fibroblast growth factor 2 (FGF2), the SET binding factor 1 (SBF1), the hook microtubule-tethering protein 3 (Hook3), the pleckstrin homology domain, and the RhoGEF domain containing G4 (PLEKHG4) (Table 1). All these targets, when aligned to predicted miRNAs, met the minimum criteria of free energy (minimum energy = 1) and score (140). PLEKHG4 polymorphisms have been related to spinocerebellar ataxia [17], a progressive-degenerative genetic disease. Also, Hook3 has been reported to interact with Pericentriolar Material 1 (PCM1) during brain development, and an imbalance in the Hook3-PCM1 interaction can cause premature depletion of the neural progenitor pool in the developing neocortex [18]. Finally, defects in the PEX26 gene can lead to a failure of protein import into the peroxisomal membrane or matrix, being the cause of several neuronal disorders, including Zellweger syndrome (ZWS), and neonatal adrenoleukodystrophy (NALD) [19, 20]. It is important to highlight that none of the predicted miRNAs were associated with every analysed ZIKV genome, nor in all isolates from the recent outbreak in Brazil. Which suggests that these predicted miRNAs are not essential for virus replication, but may improve their replication success [5]. These differences between genomes also may be related to different phenotypes of ZIKV infection, such as microcephaly in infants, Guillain—Barré syndrome [21], other symptoms similar to those of dengue and chikungunya, or asymptomatic phenotype [22].

**Table 1:** Examples of human targeting-genes to ZIKV predicted miRNAs.

Ensembl ID	Gene Symbol	Official Full Name	Cause/Effect	Reference PMID
ENSG00000215193	PEX26	peroxisomal biogenesis factor 26	Neonatal Adrenoleukodystrophy, Zellweger syndrome	17055079, 26686055
ENSG00000138685	FGF2	fibroblast growth factor 2 (basic)	Influence on cortical volume	24646670
ENSG00000100241	SBF1	SET binding factor 1	Microcephaly, mental retardation	21210780
ENSG00000168172	HOOK3	hook microtubule-tethering protein 3	Depletion of the neural progenitor pool in the developing neocortex	20152126
ENSG00000196155	PLEKHG4	pleckstrin homology and RhoGEF domain containing G4	Spinocerebellar ataxia	23572525

Interestingly, several human miRNAs known to exert an influence on the expression of genes with a known functional role in neuronal development were found to have sequence complementarity to regions in the ZIKV genome (Table 2). One of the human hsa-miR-34a miRNA targets, the Cyclin-Dependent Kinase 6 (CDK6) gene, for instance, was computationally predicted to interact with several ZIKV genomes. CDK6 is associated with the centrosome during mitosis, controlling the cell cycle division phases in neuron production [23]. Mutation in CDK6 can lead to a deficient centrosomes division, which in turns can cause autosomal recessive primary microcephaly (MCHP) [19]. There are seven well-know genes encoding centrosomal proteins that are involved in the autosomal recessive primary microcephaly (MCPH) [24], including the CDK5 Regulatory Subunit Associated Protein 2 (Cdk5rap2 or MCHP3) gene. We found a possible binding-site to the hsa-mir-324-3p, a cellular miRNA targeting Cdk5rap2 gene, in the ZIKV genomes. Equally, we found that ZIKV genomic regions can potentially bind the hsa-mir-615-3p and hsa-miR-193b-3p human miRNAs, which target the WD Repeat Domain 62 (WDR62 or MCHP2), also related to MCHP when mutated. Remarkably, a hsa-mir-21-5p miRNA complementary site was found in the genomes of ZIKV isolated from Brazil, Haiti, Martinique and French Polynesia, but not in those from Africa. This miRNA targets the MCHP4 gene, also linked to microcephaly cases. The geographic and hence historical accumulation of genomic differences may explain the recent rise of microcephaly, and this observation also corroborates the predicted pathway of transmission from Africa, through Oceania, and into Central and South America.

**Table 2:** Examples of human miRNAs with sequence complementary to ZIKV genomes.

miRNAs	Description*	Reference PMID
hsa-miR-34a	CDK6 target	23918663
hsa-mir-324-3p	Cdk5rap2/MCHP3 target	21632253 23726037
hsa-mir-15b-5p	MCPH1/BRIT1 target	12046007
hsa-mir-21-5p	MCPH4/CEP152 target	20598275
hsa-mir-335-5p	MCPH1/BRIT1 target	12046007
hsa-mir-615-3p	WDR62/MCPH2 target	20890278
hsa-miR-193b-3p	WDR62/MCPH2/ target	20890278

*All these genes have been previously related to microcephaly.

**Fig 1:**
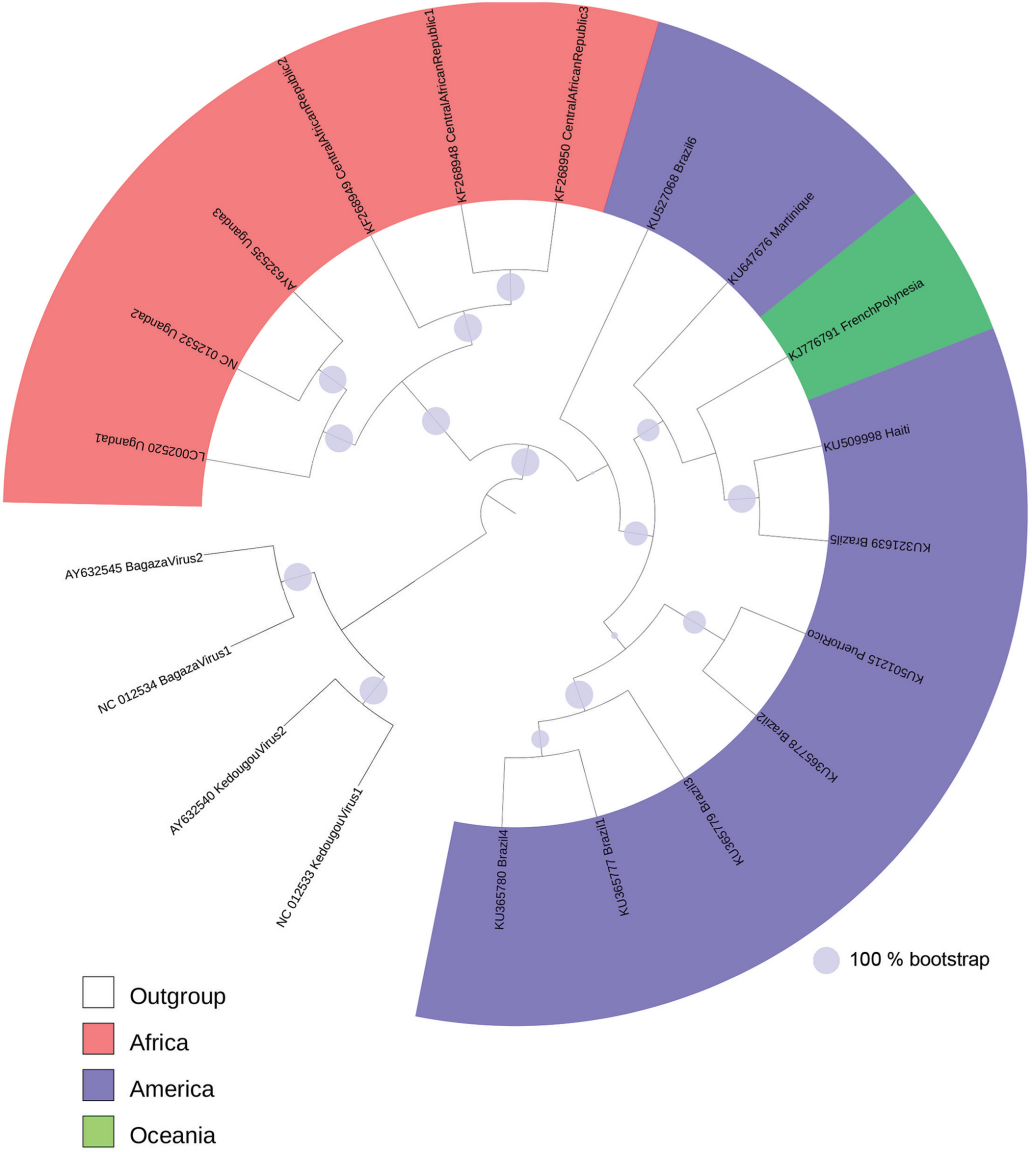
Phylogenetic analysis of all complete genomes of Zika Virus, available at GenBank (February, 2016). The GenBank accession number and the country of origin are indicated on the ZIKV branches for all strains, except for those from de out-group, where the name of the viruses is provided [Kedougou virus (NC_012533 and AY632540); Bagaza virus (AY632545 and NC_012534)]. The size of the full circles on the branches means percentage of bootstrap (100 replicates).

**Fig 2:**
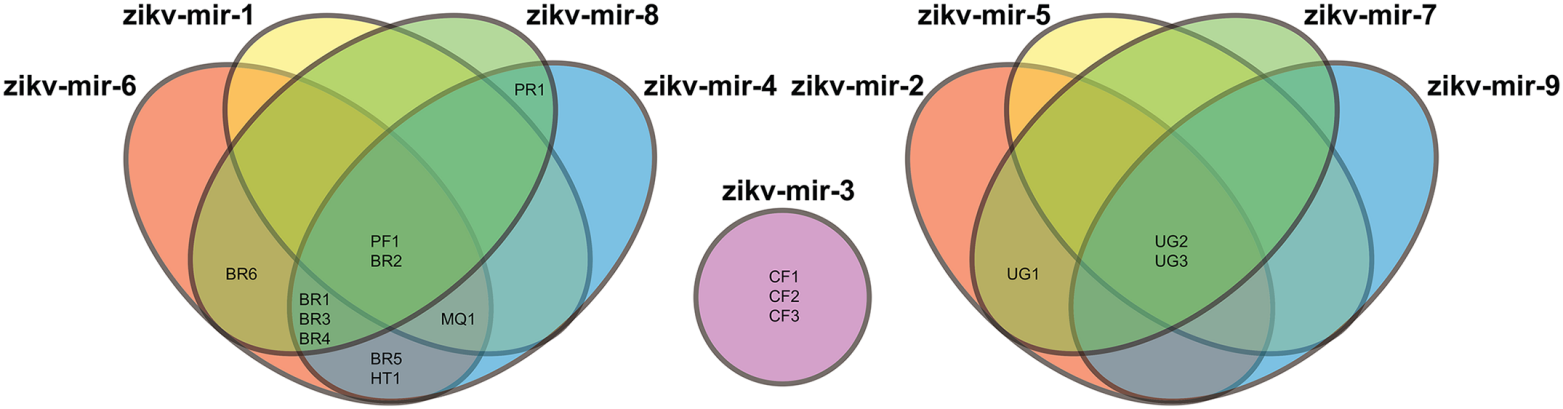
Venn diagram showing the nine different types of miRNAs predicted to be encoded by all analyzed ZIKV genomes. Brazil (BR); Central African Republic (CF); French Polinesia (FP); Haiti (HT); Martinique (MQ); Puerto Rico (PR); Uganda (UG).

To further support this, phylogenetic analysis was performed for all complete ZIKV genomes (Fig 1), which identified a cluster of strains isolated in the Americas and Oceania (derived strains), and another with the African strains (ancient strains). A third group, including two Flavivirus genotypes closely related to ZIKV, which were added as an out-group. These differences were also supported by phylogenetic analysis of only the predicted miRNAs encoded by each ZIKV strain (S1 Fig). Nine predicted miRNA types were identified, with types 1–4 being shared exclusively by derived strains and 5–8 being exclusive to ancient strains. Type 9 was only found in the genomes of strains from the Central African Republic (Fig 2). The predicted miRNAs could target a total of 14,745 human genes; 9,106 are specific to miRNAs from ancient strains, 2,840 are specific to those from derived strains, and 2,789 are shared.

Recently, ZIKV was isolated from the brain tissue of a fetus diagnosed with microcephaly [25], and two laboratory studies have provided robust evidence that ZIKV infection may cause brain defects in infants by influencing brain cell development [26, 27]. However, the mechanism by which ZIKV alters neurophysiological development remains unknown, inhibiting the development of therapeutic interventions. Our results suggest a putative influence of miRNAs on the expression of human-genes associated with the symptoms of Congenital ZIKA Syndrome. The ZIKV-CDB provides a useful knowledge base to support research targeted at mitigating the impacts of this emerging health problem [28]. ZIKV-CDB is an open-source and collaboration-based forum for sharing and identifying potential targets. The database can guide experimental investigation to elucidate the possible association between ZIKV infection and neurobiological development in infants. The ZIKV-CDB is going to be further expanded to encompass information related to others sncRNAs, as predicted by other approaches. The database will also be continuously maintained and curated by the Genomics and Computational Biology Group, FIOCRUZ/CPqRR (http://www.cpqrr.fiocruz.br).

## Supporting Information

S1 Fig Phylogenetic analysis of only the predicted miRNAs encoded by each ZIKV strain used in this study. The size of the full circles on the branches means percentage of bootstrap (100 replicates).
(PDF)

S1 Dataset FASTA file containing the precursor sequences of the nine ZIKV encoded miRNAs. (FASTA)

S2 Dataset FASTA file containing the mature sequences of the nine ZIKV encoded miRNAs. (FASTA)

S3 Dataset Tab-delimited text file containing the miRNA identifiers from ZIKV with their respective Target Ensembl Transcript, Total Score, Total Energy, Maximum Score per alignment, Maximum Energy per alignment, Strand, Length: miRNA, Length: target and Alignment positions. (TXT)

S4 Dataset Tab-delimited text file containing the miRNA identifiers from human, with their respective Target region in the ZIKV Genomes, Total Score, Total Energy, Maximum Score per alignment, Maximum Energy per alignment, Strand, Length: miRNA, Length: target and Alignment positions. (TXT)
