# Supplementary figures and images for: ZIKV – CDB: A Collaborative Database to Guide Research Linking SncRNAs and ZIKA Virus Disease Symptoms
Source: PLoS Negl Trop Dis. 2016 Jun 22;10(6):e0004817. doi: 10.1371/journal.pntd.0004817 (PMC4917180; doi:10.1371/journal.pntd.0004817)

Tree scale: 0.1

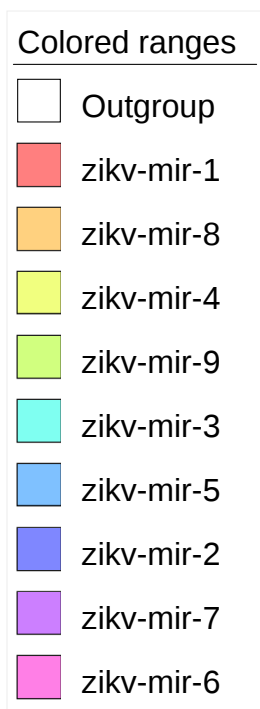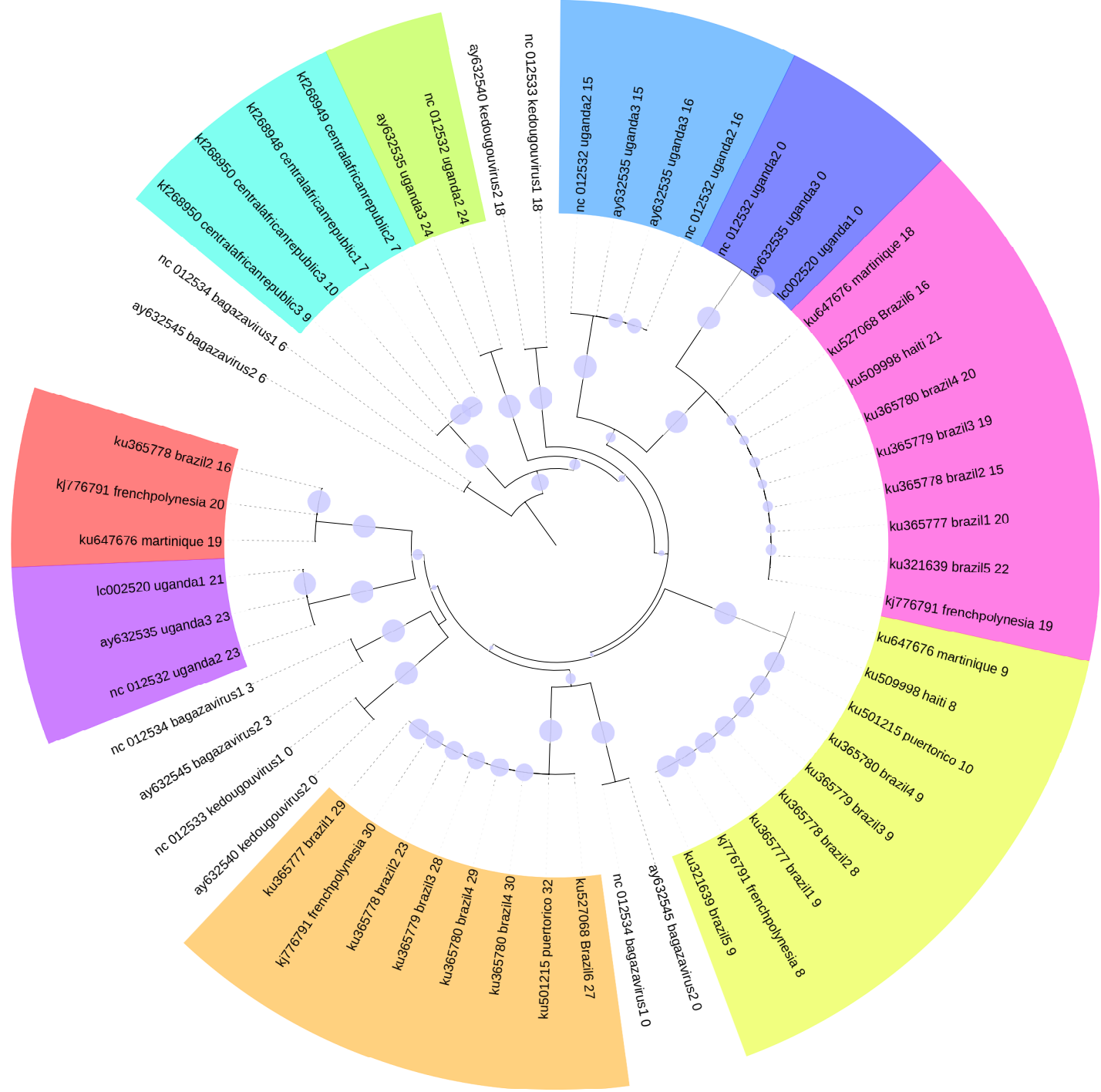

Supplement: S1 Fig — The size of the full circles on the branches means percentage of bootstrap (100 replicates). (PDF) [file pntd.0004817.s001.pdf]
